# Synthesis and Assembly of Hepatitis B Virus-Like Particles in a *Pichia pastoris* Cell-Free System

**DOI:** 10.3389/fbioe.2020.00072

**Published:** 2020-02-14

**Authors:** Alex J. Spice, Rochelle Aw, Daniel G. Bracewell, Karen M. Polizzi

**Affiliations:** ^1^Department of Chemical Engineering, Imperial College London, London, United Kingdom; ^2^The Imperial College Centre for Synthetic Biology Imperial College London, London, United Kingdom; ^3^Department of Biochemical Engineering, University College London, London, United Kingdom

**Keywords:** cell-free protein synthesis, virus-like particles, *Pichia pastoris*, synthetic biology, hepatitis B core antigen

## Abstract

Virus-like particles (VLPs) are supramolecular protein assemblies with the potential for unique and exciting applications in synthetic biology and medicine. Despite the attention VLPs have gained thus far, considerable limitations still persist in their production. Poorly scalable manufacturing technologies and inconsistent product architectures continue to restrict the full potential of VLPs. Cell-free protein synthesis (CFPS) offers an alternative approach to VLP production and has already proven to be successful, albeit using extracts from a limited number of organisms. Using a recently developed *Pichia pastoris-*based CFPS system, we have demonstrated the production of the model Hepatitis B core antigen VLP as a proof-of-concept. The VLPs produced in the CFPS system were found to have comparable characteristics to those previously produced *in vivo* and *in vitro*. Additionally, we have developed a facile and rapid synthesis, assembly and purification methodology that could be applied as a rapid prototyping platform for vaccine development or synthetic biology applications. Overall the CFPS methodology allows far greater throughput, which will expedite the screening of optimal assembly conditions for more robust and stable VLPs. This approach could therefore support the characterization of larger sample sets to improve vaccine development efficiency.

## Introduction

Cell-free protein synthesis was developed as early as the 1960s, where it was critical in determining the genetic code (Nirenberg and Matthaei, 1961). Over the decades, advances in synthetic biology are driving a renaissance in CFPS and it is emerging as a transformative platform technology (Carlson et al., 2012). This pique in interest is mainly due to the capacity to produce protein in a rapid and facile manner, providing benefits to multifarious applications. These include the design of *de novo* metabolic pathways (Hodgman and Jewett, 2012), personalized medicine (Ogonah et al., 2017) and biosensing (Pardee et al., 2016). CFPS has also shown distinct advantages in producing difficult-to-express proteins, such as those toxic to the cell *in vivo* (Katzen et al., 2005). Finally, due to the unparalleled access to the reaction environment, optimization and characterization can be achieved with an ease that far exceeds *in vivo* systems, creating opportunities for high throughput screening and rapid prototyping.

Cell-free extracts based on a variety of host organisms are continually being developed. Arguably, extracts could be prepared from any cultivatable cell-type. The most commonly utilized systems and those which are commercially available are from *Escherichia coli* (Chen and Zubay, 1983), Chinese hamster ovary cells (CHO) (Brödel et al., 2014), wheat-germ extract (WGE) (Anderson et al., 1983; Madin et al., 2000), rabbit reticulocyte lysate (RRL) (Jackson and Hunt, 1983) and insect (Sf9) cells (Stech et al., 2014). Developing new extracts is still of great interest, unlocking access to advantageous properties of the organisms from which they are derived. The available repertoire of developed CFPS extracts is still expanding and now includes HEK293 (Bradrick et al., 2013) *Saccharomyces cerevisiae* (Gan and Jewett, 2014), *BY-2* tobacco cells (Buntru et al., 2014), *Streptomyces venezuelae* (Moore et al., 2017), *Bacillus megaterium* (Moore et al., 2018), *Vibrio natriegens* (Gan and Jewett, 2014) and most recently *Pichia pastoris* (Aw and Polizzi, 2019).

*Pichia pastoris* (syn. *Komagataella* spp.) has developed over the past three decades to become the most commonly utilized protein expression system after *E. coli*, for both lab and industrial-scale protein production (Bill, 2014). The popularity of *P. pastoris* largely stems from high volumetric productivity, afforded by the ability of the organism to grow to high cell densities (Darby et al., 2012). In addition, *P. pastoris* has proven to be highly versatile, with thousands of proteins successfully synthesized to date (Ahmad et al., 2014). It has also become an important expression host for the production of VLP vaccines (Wang et al., 2016).

Virus-like particles are supramolecular protein assemblies, composed of capsid-forming proteins that self-assemble, mimicking the structure of the native viruses from which they are derived (Huang et al., 2017). VLPs are non-infectious and unable to replicate, as they lack a viral genome, removing the concerns about infectivity often associated with live-attenuated or inactivated viruses. This makes VLPs exciting potential vaccine candidates, as they also display strong immunogenicity and the induction of innate and adaptive immunity in animals and humans (Roldão et al., 2010; Crisci et al., 2012). The first VLP vaccine was commercialized in 1986 and was comprised of the hepatitis B virus surface antigen, demonstrating the efficiency and potential of VLP vaccines (Gerety, 1984). In recent years there has been a shift toward engineering increased complexity, with third generation VLPs finding applications in imaging (Manchester and Singh, 2006; Schwarz and Douglas, 2015), catalysis (Patterson et al., 2014, 2015; Maity et al., 2015) and template synthesis (Wnêk et al., 2013). Additionally, VLPs are ideal for the development of platforms for heterologous antigen display, drug encapsulation and chimeric and/or hybrid VLP vaccines (Hill et al., 2017). Advances in synthetic biology are aiding these efforts by widening the repertoire of potential antigens and improving the modularity of these platforms. Despite the attractive attributes, the use of VLPs as vaccines is still hampered by issues related to their production, such as particle integrity, yield, and purification, leading to a demand for new manufacturing approaches to address these challenges (Charlton Hume et al., 2019).

Due to the ability to quickly synthesize protein, CFPS could enable portable and expeditious manufacture of vaccines in response to emerging pathogenic threats or in resource-limited areas. Cell-free systems capable of VLP production may also find use as rapid prototyping platforms for potential VLP vaccine candidates prior to full-scale industrial manufacture or wider nanotechnology applications. Of particular interest is identifying the optimal VLP assembly conditions, which are easier to establish *in vitro* due to the open nature of the reaction environment. Greater understanding of VLP assembly may help overcome issues related to architectural heterogeneity commonly associated with inconsistent product generation. Additionally, *in vivo* expression of VLPs can cause cellular toxicity that inhibits growth, such as in the case of the toxic intermediate A2 protein, which was successfully produced and incorporated into Qβ VLPs in a cell-free system (Smith et al., 2012).

A number of CFPS systems have been utilized for the production of VLPs. Hepatitis B virus core antigen VLP has been produced in extracts derived from *E. coli* (Bundy et al., 2008) and RRL (Ludgate et al., 2016). Human papillomavirus L1 VLP has been produced in *S. cerevisiae* cell-free systems (Wang et al., 2008) and Human norovirus VLP has been produced in *E. coli* cell-free systems (Sheng et al., 2017). WGE has been used extensively for the production of VLPs derived from Hepatitis B virus (Lingappa, 1994), Hepatitis C virus (Klein et al., 2004), Human immunodeficiency virus (HIV) (Lingappa et al., 1997), and Simian immunodeficiency virus (SIV) (Dooher and Lingappa, 2004; Lingappa et al., 2005).

The history of *P. pastoris* as a robust and versatile expression host able to produce VLPs from diverse origins suggests the recently developed CFPS system could be ideally suited as a high-throughput VLP manufacturing and characterization platform. The platform could be specifically suitable for the rapid optimization and validation of DNA constructs and protein variants *in vitro*, circumventing some of the lengthy cloning procedures associated with strain development, prior to full-scale industrial manufacture *in vivo*. Additionally, recombinant protein production in the *P. pastoris* CFPS system is relatively untested. Probing the capabilities of the *P. pastoris* CFPS system for complex recombinant protein production is therefore a valuable measure of the future potential of the platform for synthesis of more complex biopharmaceuticals.

As a first step toward this goal, the wild-type Hepatitis B virus core antigen (HBc) VLP was selected as a proof-of-concept, as it is one of the best characterized model VLPs. Even though there are no clinically approved native non-chimeric HBc VLP vaccines, HBc VLPs are a popular scaffold molecule for the construction of prophylactic and therapeutic vaccine candidates, many of which are currently undergoing clinical trials (Kutscher et al., 2012). HBc VLPs are an excellent platform for foreign antigen presentation due to their high amenability to foreign insertions, and efficient self-assembly (Pumpens and Grens, 2002) and therefore have been developed for the display of foreign antigens from a vast array of pathogens (Charlton Hume et al., 2019). Some of the chimeric and/or hybrid VLPs have been approved for clinical or veterinary use; for example, against influenza (De Filette et al., 2008; Fiers et al., 2009; Kazaks et al., 2017) and malaria (Schödel et al., 1994; Sällberg et al., 2002; Nardin et al., 2004). Unlike the HBs-Ag which is an enveloped VLP, the HBc VLP is non-enveloped and therefore structurally less complex. This relative simplicity makes it a good test case for production in a cell-free system. In this study we have developed a rapid method for the production and partial purification of HBc VLPs produced in a *P. pastoris* cell-free system.

## Materials and Methods

### Media and Growth Conditions

Bacterial strains were cultured in Miller lysogeny broth (LB) medium (1% peptone from casein, 0.5% yeast extract, 1% NaCl) supplemented with 37 μg ml^–1^ Kanamycin (Sigma−Aldrich, Dorset, United Kingdom). *P. pastoris* strains were cultured in a rich yeast peptone dextrose (YPD) medium (2% peptone from casein, 1% yeast extract, and 2% dextrose) and with 350 μg ml^–1^ Geneticin (VWR, Lutterworth, United Kingdom) for selection. *P. pastoris* strains were cultured in baffled glass flasks or in 50 ml Falcon tubes at a volume of no more than 20% of the total volume of the vessel starting from an OD_600_ of 0.1.

### Strains

Bacterial recombinant DNA manipulation was carried out in *E. coli* strain NEB 5−α (New England Biolabs (NEB), Hertfordshire, United Kingdom). *P. pastoris* (syn. *Komagataella phaffi)* strain FHL1 was described previously (Aw and Polizzi, 2019).

### Plasmid Construction

The CFPS expression plasmid was generated using the Gibson DNA assembly method as described previously (Gibson et al., 2009). The pET−28b vector (Merck UK Ltd., Hertfordshire, United Kingdom) was used as a backbone. The full-length wild-type (WT) HBc core antigen (*adw* serotype) codon-optimized for *P. pastoris* and the cricket paralysis virus (CrPV) internal ribosome entry site (IRES) sequences were synthesized by GeneArt^TM^ (Thermo Fisher Scientific, Paisley, United Kingdom). In order to make the expression constructs, desired fragments were amplified with 30 bp of homology using primers purchased from Thermo Fisher Scientific using Phusion^®^ High Fidelity DNA polymerase (New England Biolabs). A Kozak sequence (GAAACG) was included in the primers, directly upstream of the coding region. This sequence was selected based on previously displayed efficacy in the production of recombinant proteins in *P. pastoris* and is recommended in the ThermoFisher PichiaPink^TM^ Expression System manual. The PCR reaction products were gel extracted using the Zymoclean^TM^ Gel DNA Recovery kit (Zymo Research Corporation, Irvine, CA, United States) prior to the assembly reaction. After confirmation of the correct plasmid assembly, a synthetic 50 bp polyA tail was generated using annealed primers and inserted into the vector by restriction cloning using *Xho*I and *Not*I (NEB). The plasmid material generated for CFPS reactions was extracted using the Qiagen Maxi Prep Kit (Qiagen, Crawley, United Kingdom).

### Crude Extract Preparation and Coupled Cell-Free Protein Synthesis

*Pichia pastoris* cells were grown and extracts were prepared as previously described (Aw and Polizzi, 2019). Briefly, overnight cultures of *P. pastoris* strain FHL1 were grown in 5 ml of YPD medium and then used to inoculate 200 ml of YPD medium to an OD_600_ of 0.1. Cell growth was allowed to proceed to an OD_600_ of 18–20 at 30°C, 250 rpm. Cells were harvested, washed, homogenized and prepared using the previously described methodology (Aw and Polizzi, 2019). Cell-free protein synthesis reactions were performed by a coupled *in vitro* transcription/translation system at a volume of 50 μL at room temperature for 3 h with no shaking as previously detailed (Aw and Polizzi, 2019).

### *In vitro* Capsid Assembly and Purification

The protocol for capsid assembly and the assembly reaction mixture was adapted from Ludgate et al., 2016. The assembly reaction mixture contained 3 μL of the product of the coupled CFPS reaction per 10 μL total assembly reaction volume and consisted of 1x restriction buffer 3 (100 mM NaCl, 50 mM Tris–HCl, 10 mM MgCl2, 1 mM dithiothreitol (DTT), pH 7.9; (NEB) supplemented with 1x complete^TM^, mini, EDTA-free protease inhibitor cocktail (Roche, Basel, Switzerland) and 1 U μL^–1^ Murine RNase inhibitor (NEB). For reactions supplemented with exogenous phosphatase, 1 U μL^–1^ of calf intestinal alkaline phosphatase (CIP; NEB) was supplemented into the assembly reaction mixture. The reaction mixtures were incubated overnight (16 h) at 37°C. At this stage, samples were then either concentrated or the purification process continued. For concentration, Vivaspin^®^ 500, 100 kDa MWCO centrifugal concentrators (Sartorius, Göttingen, Germany) were used according to the manufacturer’s instructions and samples were buffer exchanged three times with PBS to a final volume of 50 μL.

For purification, after overnight assembly, the reaction mixture was heated at 60°C for 1 h to precipitate contaminating proteins followed by centrifugation (5000 × *g*, 10 min). After centrifugation, the supernatant was collected, and solid ammonium sulfate added to 40% saturation. The mixture was incubated on a laboratory rotator for a minimum of 2 h or overnight at 4°C. The mixture was centrifuged (10,000 × *g*, 30 min) and the resulting protein pellet resuspended in a minimal volume of phosphate-buffered saline (PBS).

### SDS-PAGE Gel Analysis

Sodium dodecyl sulfate polyacrylamide gel electrophoresis (SDS–PAGE) was performed using 15% Tris–HCl SDS-PAGE gels. Protein samples were denatured by boiling for 15 min in reducing SDS sample buffer (0.0625 M Tris–HCl, pH 6.8, 2.3% (w/v) SDS, 10% (w/v) glycerol, 0.01% Bromophenol blue, 5% (v/v) β-mercaptoethanol). Molecular weight was estimated by using a pre-stained protein ladder (Pageruler, 10–170 kDa, Thermo Fisher Scientific). SDS-PAGE gels were stained using SimplyBlue^TM^ SafeStain (Invitrogen, Carlsbad, CA, United States) according to the manufacturer’s instructions.

### Dynamic Light Scattering (DLS) Measurements

Dynamic light scattering measurements were performed using a Malvern Zetasizer Nano ZS instrument (Malvern Instruments Ltd., Worcestershire, United Kingdom). The software utilized to collect and analyze the data was Zetasizer Series 7.13 from Malvern. Forty μL of each sample was analyzed in a 45 μL low-volume glass cuvette. All measurements were conducted at a controlled temperature of 25°C and for each sample 15 runs of 70 s were performed with three repetitions.

### Transmission Electron Microscopy (TEM) Analysis

A 3.4 μL sample of purified VLP solution between 0.02–0.05 mg ml^–1^ was applied to a carbon coated copper/Formvar grid and negatively stained with 2% w/v uranyl acetate, pH 4. Images were taken by a TEMCAM XF416 (TVIPS, Oslo, Norway) camera in a CM200 (Philips, Amsterdam, Netherlands) electron microscope at an acceleration voltage of 200 kV. ImageJ software (version 1.51) (Maryland, United States) was used to process the TEM images and add scale bars.

## Results and Discussion

### Synthesis, Assembly and Crude Purification of Hepatitis B Viral Capsids

The full-length wild-type HBc VLP was selected as a proof-of-concept due to extensive previous characterization and its use as a chassis molecule for novel vaccine designs (Pumpens and Grens, 2001). The icosahedral HBc VLP is composed of multiple copies of a single 21 kDa protein, the HBc protein. The WT HBc monomer can be divided into three distinct regions: an NTD (position 1–140) responsible for capsid assembly, a CTD (positions 149–183 or 185, depending on the strain) responsible for nucleic acid binding, and a linker region (position 140–149). The assembly domain contains the major immunodominant region, comprised of two α-helices forming the spikes on the particles. The full-length HBc forms dimers and the capsid itself is dimorphic, assembling into both the *T* = 3 (90 dimers) and *T* = 4 (120 dimers) icosahedral VLPs with diameters of 32 nm and 35 nm, respectively (Crowther, 1994; Roseman et al., 2012). In recent years, HBc VLPs have been used as a versatile chassis for both nanotechnology and vaccine applications (Chackerian, 2007; Ludwig and Wagner, 2007) primarily due to particle stability and integrity.

The full-length HBc gene (amino acids 1–183, codon-optimized for *P. pastoris*) was cloned into the pET−28b vector (Merck United Kingdom Ltd.) using the Gibson Assembly method (Gibson et al., 2009). *Pichia pastoris* CFPS reactions were performed to synthesize the HBc in a reaction volume of 50 μL at room temperature without shaking for 3 h.

**FIGURE 1:**
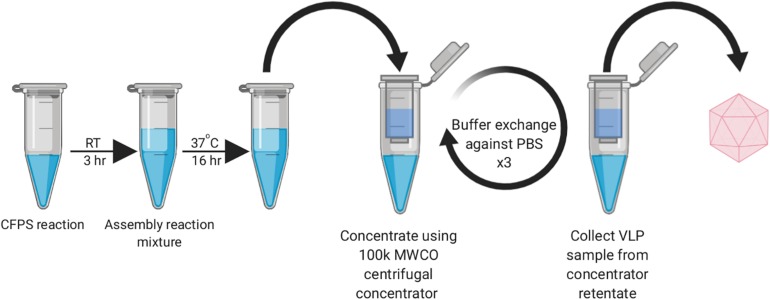
Work-flow schematic of HBc VLP synthesis, assembly and centrifugal concentrator purification. Created with biorender.com.

During our early experiments, we found that the HBc VLPs were unable to self-assemble directly in the CFPS reaction (data not shown). Previous studies have shown that HBc VLP self-assembly *in vitro* is well known to be highly dependent on monomer concentration (Lingappa et al., 2005; Ludgate et al., 2016). When the concentration of HBc dimer exceeds a critical concentration, nearly all free dimer associates into capsid (Katen and Zlotnick, 2009). The HBc concentration required for assembly *in vitro* is high (40–80 μM) at physiological salt concentrations (Wingfield et al., 1995; Tan et al., 2013), although spontaneous self-assembly of the HBc VLP has also been shown to be triggered by increasing the salt concentration (Bundy et al., 2008). Based on the previously reported yield of luciferase produced in our system (∼4.5 μM) (Aw and Polizzi, 2019), we postulated that lack of assembly was due to low dimer concentration prohibiting capsid self-assembly. In a report describing HBc VLP production in an RRL-based CFPS system, it was shown that full-length HBc VLP assembly can be achieved at lower dimer concentrations by modulating the phosphorylation state of the CTD (Ludgate et al., 2016). We therefore aimed to recreate the capsid assembly conditions from this study to ascertain whether the HBc synthesized in our system could be induced to self-assemble into capsids, even at concentrations below the self-assembly threshold. To achieve this, we designed a synthesis, assembly and purification protocol for determining the optimum particle assembly conditions (Figure 1).

In order to provide adequate time for capsid assembly, the completed CFPS reactions containing HBc were diluted into the assembly reaction mixture and incubated overnight at 37°C for 16 h. Exogenous CIP, as a broad spectrum phosphatase that can also act on protein, was supplemented into selected samples to dephosphorylate the CTD of the HBc in order to modulate capsid assembly (Ludgate et al., 2016). Since the CTD undergoes non-specific RNA binding, which is modulated by its phosphorylation state, inclusion of an RNase inhibitor in the assembly reaction mixture was also essential to preserve RNA integrity (Ludgate et al., 2016). It is highly probable therefore that dephosphorylation of the CTD promotes non-specific interactions with nucleic acids, facilitating capsid assembly. It has been previously shown that a positively charged CTD interacts with negatively charged nucleic acids to enable capsid assembly at 5 nM (Klein et al., 2004) and the removal of the phosphate may contribute to increasing the local positive charge.

After incubation, centrifugation was conducted to remove aggregates and the supernatant was applied to a 100 kDa MWCO centrifugal concentrator and buffered-exchanged three times against PBS. A high MWCO membrane is frequently used in HBc VLP production as a concentration and rudimentary purification step (Kazaks et al., 2017; Wetzel et al., 2018).

**FIGURE 2:**
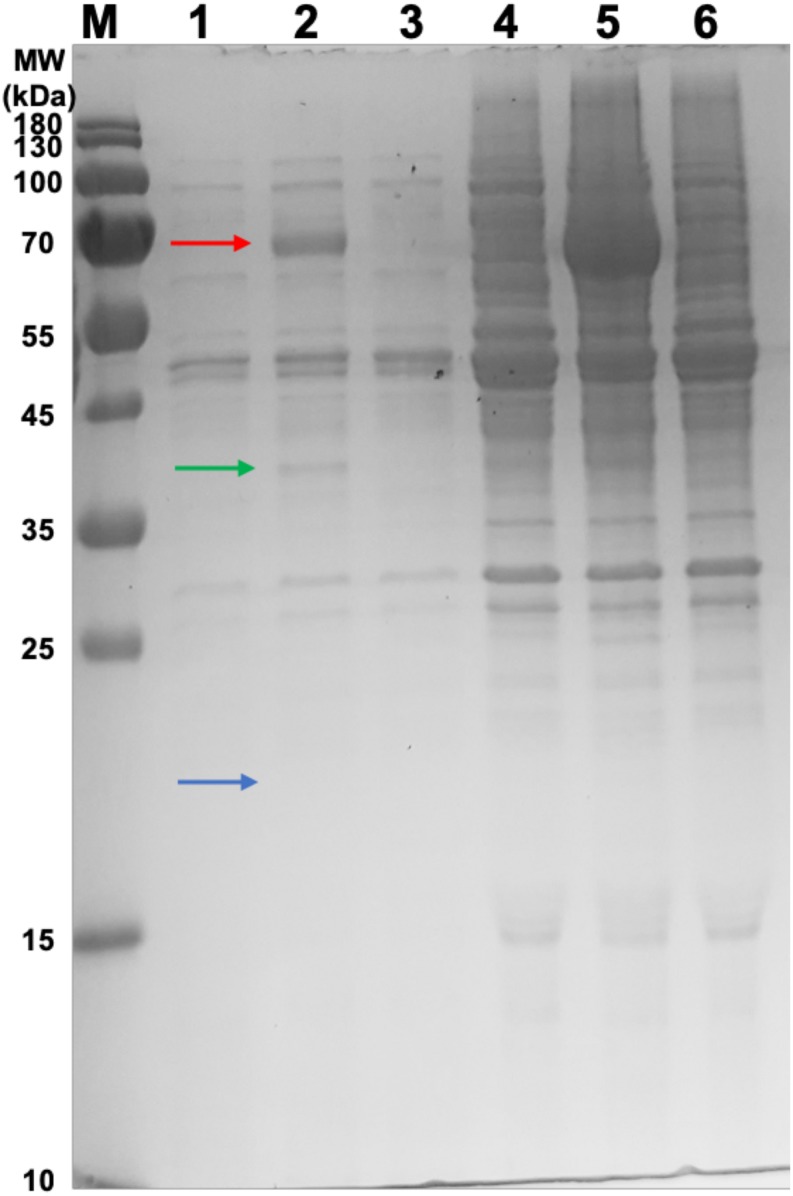
SDS-PAGE gel of samples from the concentration of CFPS products after incubation in the assembly reaction mixture and associated flowthrough. Lane M: PageRuler pre-stained protein ladder. Lane 1, CFPS HBc retentate; Lane 2, CFPS HBc (+CIP) retentate; Lane 3, CFPS NC (no HBc template) retentate; Lane 4, CFPS HBc flowthrough; Lane 5, CFPS HBc (+CIP) flowthrough; Lane 6, CFPS NC (no HBc template) flowthrough. Red arrow indicates CIP (∼70 kDa). Green arrow indicates HBc dimer (∼40 kDa). Blue arrow indicates absence of band for HBc monomer (21 kDa).

Samples were taken from the retentate of the concentrator and the resultant flow-through for SDS-PAGE analysis (Figure 2). The HBc monomer has a molecular weight of 21 kDa, but no band of this size was observed across all samples. A band was, however, observed at approximately 40 kDa for the CIP-treated samples (green arrow) which could be indicative of the HBc dimer (42 kDa). Presence of a band at the expected size of the dimer in the CIP-treated samples suggested HBc VLP assembly, though further confirmation was required. It is highly likely that the HBc monomer is not visible in the flowthrough of the untreated HBc samples due to dilution. Based on previous concentrations of luciferase produced and the molecular weight of HBc, the concentration is insufficient to meet the criteria for SDS-PAGE detection with SimplyBlue^TM^ SafeStain (Invitrogen). It is worth noting that the other prominent band found in the CIP-treated samples at approximately 70 kDa is the CIP monomer (red arrow), which is added at very high concentrations.

### Characterization by DLS

**FIGURE 3:**
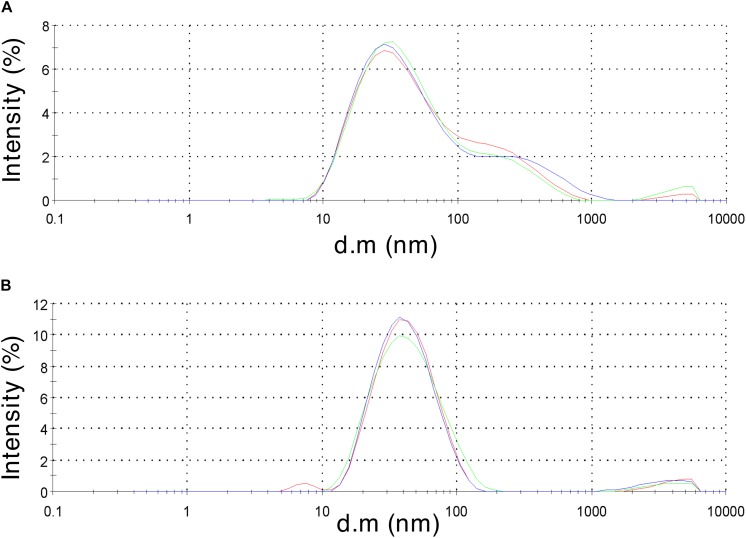
Dynamic light scattering measurements to characterize particle size distribution of the assembled HBc VLPs. **(A)** HBc VLPs obtained from incubation in assembly buffer in the absence of CIP (untreated assembly) **(B)** HBc VLPs obtained from incubation in assembly buffer supplemented with CIP (CIP-treated assembly). Samples are displayed as intensity percentage with three technical replicates.

To validate HBc VLP assembly, retentate samples taken after centrifugal concentration were subjected to morphological analysis. Samples of 40 μL were obtained from the concentration step and transferred to a 45 μL low-volume glass cuvette. Using DLS, the diffusion of the particles moving under Brownian motion was measured and the particles sizes of VLPs calculated. Both the CIP-treated and untreated HBc samples gave a positive result from the DLS characterization (Figure 3). The untreated HBc sample (Figure 3A) displayed a Z-average particle size of 36.89 nm. Considering our initial hypothesis that VLPs would be unable to assemble as a result of low dimer concentration, this was an unexpected result. It is possible that the concentration of monomer produced in our CFPS system is sufficient for capsid self-assembly, but only when subjected to the conditions provided by the assembly buffer. The beneficial conditions promoting capsid assembly could be the increased salt concentration of the assembly buffer, which was previously shown to trigger capsid self-assembly (Bundy et al., 2008), the inclusion of RNase inhibitor, which preserves RNA integrity thereby facilitating co-localization of free dimer, or a combination of the two. The CIP-treated HBc sample (Figure 3B) displayed a Z-average particle size of 37.97 nm. Both observed Z-average particle sizes are close to the reported size of the *T* = 4 HBc VLP (∼35 nm) (Crowther, 1994; Zlotnick et al., 1996). The *T* = 4 capsid has been reported as the most abundant product of WT HBc produced by *in vitro* self-assembly (∼95%) (Zlotnick et al., 1999).

### Characterization by TEM

**FIGURE 4:**
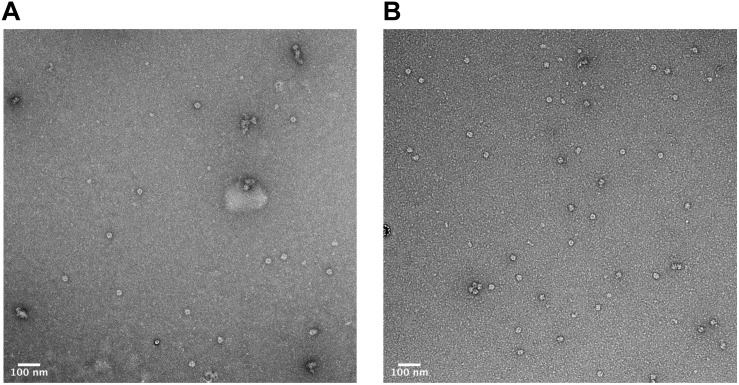
Characterization of HBc VLPs obtained from assembly and crude purification using transmission electron microscopy (TEM). **(A)** HBc VLPs obtained from incubation in assembly buffer in the absence of CIP (untreated assembly) **(B)** HBc VLPs obtained from incubation in assembly buffer supplemented with CIP (CIP-treated assembly). Scale-bar size: 100 nm.

In order to verify that the products of our rapid synthesis, assembly and purification protocol contained correctly formed HBc VLPs, TEM with 2% uranyl acetate negative staining was used (Figure 4). The average diameter for the untreated HBc VLPs was 29.3 nm (SD = 1.4 nm, *n* = 13) and from the CIP-treated HBc VLPs the average diameter was 28.9 nm (SD = 1.8 nm, *n* = 19). The average size of particles from both samples correlate with previously reported diameters of 30.7 nm from HBc VLPs produced using *in vitro* methods (Bundy et al., 2008) and 30 nm from *in vivo* methods (Zlotnick et al., 1996). Interestingly, a greater number of fully assembled particles were visible in the CIP-treated sample relative to the untreated sample. This could explain why a faint band with a size corresponding to the dimer was visible in the SDS-PAGE analysis for the CIP-treated sample (Figure 2, Lane 2), but no band corresponding to the expected size of the monomer or dimer was observed for the untreated samples (HBc) (Figure 2, Lane 1). As previously discussed, dephosphorylation of the CTD likely boosts capsid assembly by promoting non-specific interactions with nucleic acids, facilitating capsid self-assembly. Despite this, there was no great observable difference in average particle size or morphology with both samples still displaying a proportion of partially assembled or disassembled particles, in addition to other smaller protein contaminants. It is possible the smaller contaminants are HBc monomer, CIP or host-cell protein carried through the purification. The presence of host-cell contaminants correlates with the SDS-PAGE analysis (Figure 2) suggesting further development of the purification protocol was needed.

### Heat-Treatment and Ammonium Sulfate Purification of Assembled HBc VLPs

**FIGURE 5:**
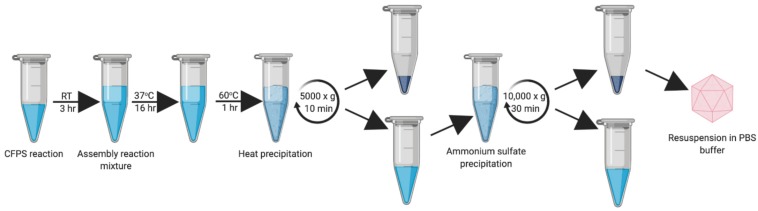
Updated work-flow schematic of HBc VLP synthesis, assembly, heat treatment and ammonium precipitation. Created with biorender.com.

Once the successful synthesis and assembly of HBc VLPs was demonstrated, the next aim was to develop an improved methodology for VLP purification (Figure 5). We decided to develop this process using the CIP-treated samples, as the TEM characterization indicated an increased average number of particles relative to the untreated samples. In order to keep in line with creating a simple and rapid purification process, we strived to avoid more time-consuming purification steps, such as chromatography, and those that are costly and poorly scalable for production purposes, such as ultracentrifugation. It has been reported that an efficient method of removing host-cell protein contaminants is the inclusion of a heat treatment step which is both fast and simple (Ng et al., 2006; Lee et al., 2015; Li et al., 2018). As fully-assembled HBc VLP has been shown to be highly thermostable (Ng et al., 2006; Lee and Tan, 2008), heat-labile host-cell proteins can be denatured and precipitated from solution. Toward this end, we removed the centrifugal concentrator step and included a 1 h incubation at 60°C after the initial assembly reaction in order to remove protein contaminants and improperly assembled HBc VLPs. After heating, the reaction mixture was subjected to centrifugation and the supernatant collected for ammonium sulfate precipitation.

Ammonium sulfate precipitation is a well-established method for separation of HBc VLPs and is widely used as a preparatory purification step (Palenzuela et al., 2002). Particles were precipitated by addition of solid ammonium sulfate to 40% saturation (Yang, 2005) followed by incubation at 4°C on a rotator for a minimum of 2 h. The reaction was then centrifuged, and the resultant precipitate resuspended in a minimal volume of PBS, thereby purifying and concentrating the HBc VLPs.

**FIGURE 6:**
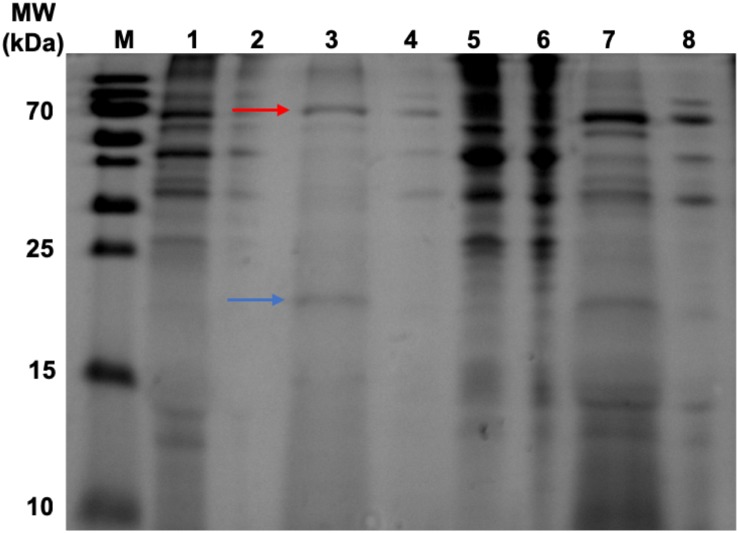
SDS-PAGE gel displaying samples taken during heat-purification and ammonium sulfate precipitation post assembly reaction. Lanes 1–4 have undergone heat treatment then ammonium sulfate precipitation. Lanes 5–8 have undergone heat treatment only. Lane M, PageRuler pre-stained protein ladder; Lane 1, HBc (+CIP) supernatant after heat treatment and ammonium sulfate precipitation; Lane 2, Negative control (NC) (+CIP) supernatant after heat treatment and ammonium sulfate precipitation; Lane 3, HBc (+CIP) precipitate after heat treatment and ammonium sulfate precipitation; Lane 4, NC (+CIP) precipitate after heat treatment and ammonium sulfate precipitation; Lane 5, HBc (+CIP) precipitate after heat treatment only; Lane 6, NC (+CIP) after heat treatment only; Lane 7, HBc (+CIP) supernatant after heat treatment only; Lane 8, NC (+CIP) supernatant after heat treatment only. The blue arrow indicates the expected band size of the HBc monomer (21 kDa). The red arrow indicates the expected band size of the CIP protein (70 kDa).

**FIGURE 7:**
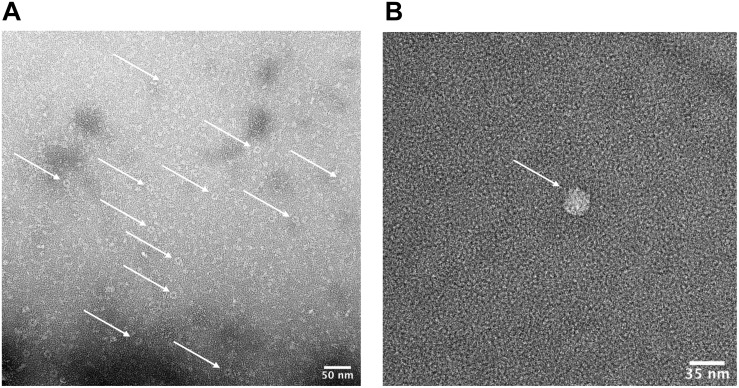
Characterization of HBc VLPs obtained from a heat-treatment and ammonium sulfate purification method. Images generated using transmission electron microscopy (TEM). **(A)** Samples were prepared directly from material resuspended after ammonium sulfate precipitation. The arrows indicate a number of fully formed HBc VLPs. Scale-bar size: 50 nm. **(B)** Sample was prepared at a lower concentration of 5 μg ml^–1^. Staining was preceded by three immediate washes in filtered distilled water after the sample was applied to the grid. The arrow indicates a fully formed HBc VLP. Scale-bar size: 35 nm.

To validate the efficacy of our new purification method, samples were taken from the precipitate and supernatant from each step in the purification process for SDS-PAGE analysis (Figure 6). The initial heat treatment step, included to denature and precipitate less thermostable host-cell proteins, appears to remove a large number of contaminants as shown in Lanes 5 and 6 which correspond to the resuspended precipitates from the heat treatment of the HBc assembly reaction containing CIP and negative control. Heat treatment alone does result in sufficient purification to detect the presence of a band at approximately 20 kDa in the supernatant of the HBc assembly reaction (Lane 7), which correlates to the expected size of the HBc monomer (21 kDa). There are still a number of other protein contaminants present, including a band at 70 kDa indicative of CIP. CIP cannot be heat inactivated for removal unless heated to 80°C, which would cause dissociation of the assembled HBc VLPs (Li et al., 2018). Despite the presence of CIP, we note that much of the CIP is removed during the improved purification process. For example, in Figure 7 the CIP band in lane 7 (representing CIP remaining in the sample after heat treatment alone) is much more intense relative to that of the HBc monomer than the CIP band in lane 3 (representing CIP remaining after both heat treatment and ammonium sulfate precipitation) relative to the HBc monomer.

To improve the purity of the VLPs, a number of possible strategies could be employed. The concentration of CIP used in this study was selected based on that previously reported (Ludgate et al., 2016); however, a lower concentration may still be sufficient for CTD dephosphorylation to promote assembly and would reduce the amount of CIP contaminant present in the sample after purification of the HBc VLPs. Alternatively, a heat-labile alkaline phosphatase such as one derived from Shrimp (Nilsen et al., 2001) could be used instead of CIP (Mehta et al., 2009; Crawley et al., 2011), enabling its irreversible removal from the sample via heat inactivation at 65°C. This would enable removal of the phosphatase alongside the heat-labile host-cell proteins during the heat inactivation step of the purification procedure. Finally, a hexahistidine, FLAG, or other affinity purification tag could be fused to recombinantly produced CIP allowing its removal with a single chromatography step.

After adding the additional ammonium sulfate precipitation step, there are two prominent bands remaining in the HBc assembly reaction containing CIP sample (Lane 3) at the expected band size of the HBc monomer (blue arrow) and CIP protein (red arrow). It is possible that some of the CIP protein remains bound to HBc during the purification process or it may become encapsulated as the VLP forms, leading to its persistence. For example, it was recently observed that an influenza VLP contained an internal component density postulated to be encapsulated cellular components (McCraw et al., 2018). The CIP protein is also visible in the negative control sample containing CIP (Lane 4), along with other higher molecular weight contaminants.

The presence of a single band indicative of the HBc monomer in Figure 6 compared to the observation of a band indicative of the HBc dimer in Figure 2 could be due to a combination of factors. Firstly, there is large disparity in the total protein concentration of the samples with those in Figure 2 having a much greater total protein concentration compared to those in Figure 6. It is possible, therefore, that the amount of SDS and/or beta-mercaptoethanol present in the sample to solubilize and fully denature the amount of protein was insufficient. Secondly, the HBc dimer in Figure 2 could be due to re-dimerization of the HBc monomer between sample preparation and loading. It has previously been reported that disulfide re-oxidation can occur during SDS-PAGE (Kumar et al., 1993; Grigorian, 2005) and both the HBc monomer and dimer have been previously observed to co-occur (Storni and Bachmann, 2004).

The total protein concentration of the purified HBc VLP sample was obtained by measuring protein absorbance at 280 nm (A280) and found to be 68 μg ml^–1^. Densitometry analysis conducted using the ImageJ software was used to estimate the yield of HBc VLP in our system at 6.4 μg ml^–1^. This is a slight improvement on the reported yield of HBc VLP of 4 μg ml^–1^ previously produced in a WGE system (Lingappa et al., 2005). The yield is currently considerably lower in our system compared to production of HBc VLP *in vivo*, with reported yields of assembled HBc VLP in *P. pastoris* up to 3 mg per 1 g wet cell weight (Freivalds et al., 2011).

### Characterization of Purified HBc VLPs by TEM

We next sought to determine whether the product of our improved purification protocol contained correctly formed HBc VLP. To achieve this, TEM with 2% uranyl acetate negative staining was performed (Figure 7A). A number of fully formed HBc VLPs can be observed with an overall increase in the uniformity of their morphology compared to Figure 4. This indicates removal of partially assembled or disassembled HBc VLPs in the developed purification scheme relative to the initial TEM analysis using the crude purification method (Figure 4). A number of misfolded or aggregated proteins still persist in the sample, however. Based on the SDS-PAGE analysis in Figure 6, we postulate that these are likely to be aggregates of CIP, as only fully formed VLPs remain in the supernatant after heat treatment (Ng et al., 2006). An additional TEM grid was prepared at a lower sample concentration of 5 μg ml^–1^. Staining was followed by three washes in filtered distilled water for 1 min each in order to attain single particle imaging for accurate size estimation (Figure 7B). The wash steps were included in the staining procedure to remove excess uranyl acetate stain and led to a reduction in stain permeation of the HBc VLPs (Figure 7B). The observed diameter for the displayed particle was approximately 29.5 nm. The reported size correlates with previously reported diameters from HBc VLP produced using *in vitro* methods (Bundy et al., 2008), *in vivo* methods (Zlotnick et al., 1996) and our previously shown assembled particle diameter of 29.4 nm using the crude purification method.

## Conclusion

Cell-free protein synthesis is an incredibly powerful and versatile tool in synthetic biology, which can be applied in myriad ways. We have generated a rapid HBc VLP synthesis, assembly and purification scheme demonstrating the potential of the *P. pastoris* CFPS system for the production of complex supramolecular assemblies. This approach could enable ultra-scale-down rapid prototyping of protein variants for novel vaccine technologies. Additionally, by exploiting the open nature of the reaction environment, the conditions for assembly can be optimized, alleviating the current challenges of structural heterogeneity in VLP manufacture. We plan to expand on this work, further testing the capabilities of the system by producing VLPs of increased complexity. In future, we envision increased effectiveness in response to emerging pathogenic threats, through high-throughput vaccine development approaches.

## Data Availability Statement

All datasets generated for this study are included in the article/supplementary files.

## Author Contributions

AS designed and performed the experiments. RA and KP helped to design the experiments. KP and DB helped to conceive the study. All authors helped to draft the manuscript and read and approved the final manuscript.

## Conflict of Interest

The authors declare that the research was conducted in the absence of any commercial or financial relationships that could be construed as a potential conflict of interest.

## Abbreviations

CFPS: cell-free protein synthesis
CIP: calf-intestinal alkaline phosphatase
CTD: C-terminal domain
DLS: dynamic light scattering
HBc: hepatitis B core antigen
MWCO: molecular weight cut-off
NTD: N-terminal domain
OD_600_: optical density at 600 nm
TEM: transmission electron microscopy
VLP: virus-like particle.
